# A Novel Treatment Strategy for Unresectable Locally Recurrent Rectal Cancer—Upfront Carbon-Ion Radiotherapy Followed by Surgical Resection of the Irradiated Intestines

**DOI:** 10.3390/cancers17132230

**Published:** 2025-07-03

**Authors:** Kei Kimura, Hirotoshi Takiyama, Shigeru Yamada, Kazuma Ito, Mizuki Koba, Ayako Imada, Jihyung Song, Kozo Kataoka, Takako Kihara, Ikuo Matsuda, Naohito Beppu, Yuki Horio, Kazuhiro Kitajima, Motoi Uchino, Hiroki Ikeuchi, Masataka Ikeda

**Affiliations:** 1 Division of Lower G.I., Department of Gastroenterological Surgery, School of Medicine, Hyogo Medical University, Nishinomiya 663-8501, Japan; ka-itou@hyo-med.ac.jp (K.I.); mi-koba@hyo-med.ac.jp (M.K.); ay-imada@hyo-med.ac.jp (A.I.); ch-son@hyo-med.ac.jp (J.S.); ko-kataoka@hyo-med.ac.jp (K.K.); n_beppu@yahoo.co.jp (N.B.);; 2 QST Hospital, National Institutes for Quantum Science and Technology, Chiba 263-8555, Japan; takiyama.hirotoshi@qst.go.jp (H.T.); yamada.shigeru@qst.go.jp (S.Y.); 3 Department of Surgical Pathology, Hyogo Medical University, Nishinomiya 663-8501, Japan; ta-kihara@hyo-med.ac.jp (T.K.); matsudai@hyo-med.ac.jp (I.M.); 4 Division of Inflammatory Bowel Disease Surgery, Department of Gastroenterological Surgery, School of Medicine, Hyogo Medical University, Nishinomiya 663-8501, Japan; yu-horio@hyo-med.ac.jp (Y.H.); uchino2s@hyo-med.ac.jp (M.U.); ikeuci2s@hyo-med.ac.jp (H.I.); 5 Department of Radiology, School of Medicine, Hyogo Medical University, Nishinomiya 663-8501, Japan; ka-kitajima@hyo-med.ac.jp

**Keywords:** locally recurrent rectal cancer, carbon-ion radiotherapy, rectal cancer

## Abstract

**Background/Objectives:** Carbon-ion radiotherapy (CIRT) is a promising treatment option for unresectable locally recurrent rectal cancer (LRRC). However, CIRT is contraindicated in cases where recurrent tumors are attached to the intestine. To address this limitation, we developed a novel treatment strategy involving curative-dose CIRT to recurrent tumors, including the adjacent intestine, without dose constraints, followed by surgical resection of the irradiated intestine. This study aimed to assess the feasibility of this approach. **Methods:** Patients were eligible for this study if the distance between the unresectable recurrent tumor and the adjacent intestines was less than 3 mm. Between 2019 and 2023, twelve patients were enrolled. CIRT was administered at curative doses of 70.4 or 73.6 Gy (relative biologic effectiveness (RBE)), including the adjacent intestines, without dose constraints. Surgical resection was not intended to excise the tumor itself, but was performed solely to remove the irradiated intestines. Irradiated intestine resection was planned within eight weeks after the completion of CIRT. **Results:** All patients completed the scheduled treatment course. The median interval between completing CIRT and surgery was 4 (3–8) weeks. No patients experienced acute AEs related to CIRT. Regarding late AEs, two patients developed Grade I sciatic neuralgia, and one patient developed Grade III neuralgia. We considered this symptom, which later resulted in a limp in his left leg, acceptable because this patient could ambulate with assistance. Clavien–Dindo Grade III postoperative complications occurred in one patient. The median follow-up duration was 40 (20–60) months. One patient was diagnosed with in-field recurrence, and three patients were diagnosed with out-of-field recurrence. These patients received reirradiation with CIRT. Four patients experienced lung recurrence, and one patient died from rectal-cancer-specific causes. **Conclusions:** This novel treatment strategy may provide favorable outcomes for patients with unresectable LRRC. This approach can be applied to the currently accepted indications for CIRT, and we believe that CIRT is a feasible treatment option for future patients.

## Simple Summary

R1/R2 resection for locally recurrent rectal cancer (LRRC) has a poor prognosis, comparable to that of nonsurgical treatments. Given the high invasiveness and its association with quality of life deterioration, carbon-ion radiotherapy (CIRT) is considered a feasible treatment option for unresectable LRRC in Japan. CIRT offers a narrow dose distribution that spares normal organs and increases the rate of cell apoptosis. However, LRRC occurs within the narrow pelvis and often adheres to adjacent intestines, making CIRT unfeasible. To maintain a safe distance between the tumors and intestines, surgical spacer placement has been performed; however, this intervention carries the risk of secondary tumor dissemination and iatrogenic injury to the intestinal tract. In this study, we evaluated the feasibility of a novel treatment strategy, namely, upfront CIRT followed by surgical resection of the irradiated intestines. Although the study had a small sample size, our results suggest that this approach can be performed safely.

## 1. Introduction

For locally recurrent rectal cancer, surgical resection is the first choice of treatment and often requires the combined resection of adjacent organs [1,2,3,4,5]. However, the complicated pelvic anatomy of the lateral pelvic compartments limits the indications for surgical resection and makes it challenging to achieve R0 resection [6,7,8]. Recently, carbon-ion radiotherapy (CIRT) has received attention as a promising treatment option for unresectable locally recurrent rectal cancer (LRRC) in Japan [9,10,11,12]. It is characterized by a narrow dose distribution that protects healthy organs and includes frequent DNA double-strand breaks, leading to a relatively high rate of cell apoptosis. Unfortunately, CIRT is contraindicated for patients with recurrent tumors attached to the surrounding intestines due to radiation-induced adverse events (AEs), such as ulcers, bleeding, or intestinal perforation [13,14]. For unresectable LRRC near the intestine, we place a surgical spacer between the tumor and adjacent intestines to allow adequate irradiation via CIRT [15]. In prostate cancer treatment, spacer placement ensures an effective margin from the rectum [16,17,18,19]. Local recurrent tumors typically occur within the narrow pelvis, which often adhere to the adjacent intestines, making CIRT unfeasible, because performing a spacer placement procedure increases the risk of secondary tumor dissemination and iatrogenic intestinal injury. The use of spacers is not recommended in cases involving bowel resection due to the increased risk of infection. Therefore, alternative treatment approaches involving CIRT are needed. Here, we demonstrate a novel treatment strategy involving upfront curative-dose CIRT targeted at recurrent tumors to ensure curability and address the potential risk of severe adverse events (AEs) by planned surgical resection of the irradiated intestine. This study aimed to evaluate the safety of our new combination treatment.

## 2. Materials and Methods

### 2.1. Patients

**Figure 1:**
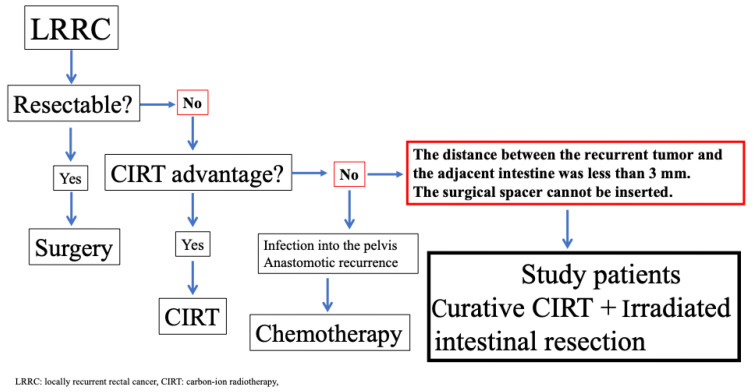
Patient eligibility criteria.

Patients were selected from the locally recurrent rectal cancer database of Hyogo Medical University Hospital between April 2019 and December 2023. This study was approved by the institutional review board of Hyogo Medical University (No. 4440). LRRC was defined as recurrence in the pelvis after prior treatment, with patients required to have been disease-free for at least six months between the initial surgery and the diagnosis of LRRC. All computed tomography (CT) and magnetic resonance imaging (MRI) images were assessed by gastrointestinal radiologists and colorectal surgeons. Our treatment strategy for LRRC patients is shown in Figure 1. CIRT was recommended for patients with LRRC if radical surgery was contraindicated, such as in cases of unresectable distant metastasis, unfit medical status, sacral involvement above the S2 vertebra, invasion into the lateral pelvic compartments extending beyond the internal obturator muscles, or when patients declined extensive surgery [20]. On the other hand, CIRT is contraindicated for patients with recurrent tumors located less than 3 mm from the adjacent intestine [21]. In such cases, spacer placement is generally indicated to ensure an appropriate distance from the recurrent tumor [15]. However, in patients with LRRC, spacer placement is contraindicated when preoperative imaging suggests severe adhesion between the tumor and the adjacent intestines, as mobilizing the adjacent intestine may increase the risk of tumor dissemination or intestinal injury. The eligibility criteria for this study were that the distance between the unresectable recurrent tumor and the adjacent intestine was less than 3 mm and that the surgeons could not insert the surgical spacer. Patients with anastomotic recurrence or LRRC invading the bowel lumen were excluded.

### 2.2. Treatment

Treatment strategies for LRRC were defined for each patient, following an interdisciplinary online meeting between Hyogo Medical University and the National Institutes for Quantum Science and Technology (QST) Hospital. CIRT was administered to the enrolled patients using the methods described by Yamada et al. at QST Hospital [10,22]. The treatment dose for CIRT was set at 73.6 gray relative biologic effectiveness (Gy (RBE)) for patients without a history of prior irradiation or at 70.4 Gy (RBE) for patients with a history of previous irradiation to the pelvic area, and it was delivered in 16 fractions without concurrent chemotherapy [21]. The treatment was performed daily, four days a week. The target field included the gross tumor volume and the surrounding clinical target volume with a 10 mm margin to the tumor. Therefore, the field partially included the adjacent intestines in the planned irradiation field. Within approximately eight weeks after the completion of CIRT, intestinal tracts irradiated with more than 46 Gy were considered for resection [23]. The extent of the irradiated intestines was carefully discussed at the two hospitals.

A key concept of this study was that the surgery was not intended for tumor excision, but for the removal of irradiated intestines that carried a high risk of severe complications such as massive bleeding, obstruction, or perforation. The LRRC itself was treated with curative intent through CIRT and was not included in surgical resection. During surgery, we marked the extent of intestinal resection using crystal violet following the distribution map by identifying local adhesions of the intestine on the pelvis and the small intestine dropping down into the pelvic cavity before shifting the patient to the surgical position. When the neorectum, including the anastomotic site, was resected, the LRRC itself remained in the pelvis, and only the irradiated intestines were removed. In addition, careful attention was required to prevent intestinal injury, as it could lead to severe postoperative pelvic sepsis. Resection of the neorectum was minimized, while a wider resection of the small intestine was performed when it adhered to the tumor, since the irradiated area of the small intestine was often unclear. If the recurrent tumors were firmly fixed to the mesentery, only the irradiated intestinal wall was resected without dissecting the mesentery. Finally, an omental pedicle flap was placed between the recurrence site and the intestine to enable reirradiation in cases of tumor re-recurrence [24,25].

### 2.3. Follow-Up

Acute and late AEs of CIRT were defined according to the National Cancer Institute-Common Toxicity Criteria for Adverse Events version 5.0 [26]. Acute AEs were defined as toxicity persisting within 90 days after the completion of CIRT. Late AEs were defined as the presence of toxicity observed 3 months or later after irradiation. Severe AEs of CIRT were defined as those that were Grade 3 or higher [27]. Postoperative complications were assessed using the Clavien–Dindo classification [28]. All resected samples were investigated by specialized GI pathologists.

For follow-up, the standard protocol included carcinoembryonic antigen (CEA) and carbohydrate antigen (CA19-9) measurements every three months and thoracoabdominal computed tomography (CT) once every six months for five years after surgery. If suspected systemic recurrence was found, further examination to ensure an accurate diagnosis was performed using magnetic resonance imaging (MRI) or positron emission tomography–CT, depending on the site of recurrence. Regarding local re-recurrence, we also defined “in-field recurrence” as an enlarged recurrent tumor inside the planning target volume and “out-of-field recurrence” as an enlarged recurrent tumor outside the planning target volume.

Clinical data, including patient characteristics, previous history of rectal cancer, CIRT details, irradiated intestine resection details, postoperative complications, histopathological findings, and prognosis, were collected. Survival and disease recurrence were described to determine oncologic outcomes.

## 3. Results

**Figure 2:**
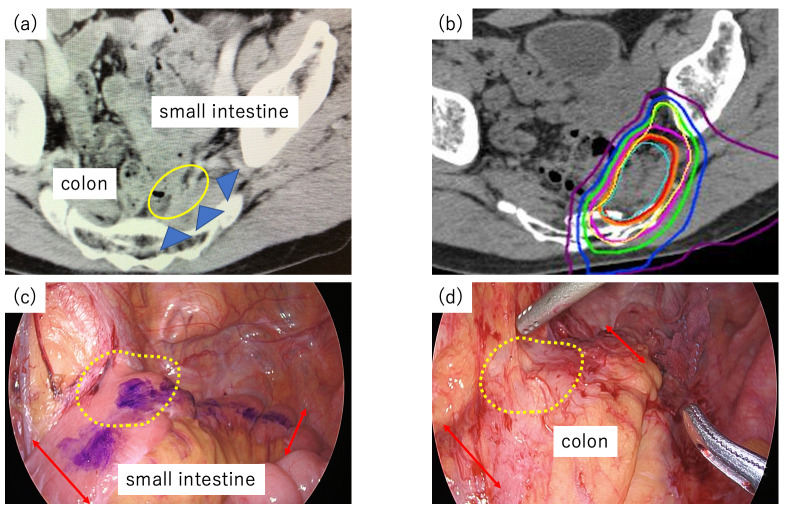
(**a**) Computed tomography scans showing locally recurrent tumors (yellow circles and blue arrowheads). (**b**) Depth-dose distribution of carbon-ion beams in recurrent rectal cancer patients. Areas encircled with orange, magenta, green, dark-marine, and violet lines indicate more than 90%, 80%, 50%, 30%, and 10% energy accumulation, respectively. Yellow lines indicate areas of interest for irradiation. (**c**) The extent of small intestine resection. Surgical resection of the irradiated small intestine with ≥46 Gy (RBE) was planned. (**d**) The extent of neorectum resection. Surgical resection of the irradiated neorectum of ≥46 Gy (RBE) was planned.

A total of twelve patients were included in this study; the patient characteristics are summarized in Table 1. Among these 12 patients, 10 were male, and the median age was 64 years (range, 38–78 years). Curative surgery was contraindicated in ten patients because of the necessity of bilateral S1 nerve resection, in one patient with lateral recurrence infiltrating beyond the internal obturator muscles, and in one patient who declined extensive surgery because of intolerable deterioration in postoperative quality of life. Details of CIRT for patients who underwent this novel treatment are shown in Table 2. All patients completed the scheduled treatment course. Nine patients were administered 73.6 Gy (RBE) in 16 fractions; on the other hand, three patients with prior pelvic X-ray radiotherapy were administered 70.4 Gy (RBE) in 16 fractions. Figure 2 shows a case of LRRC adjacent to the neorectum and small intestine.

**Figure 3:**
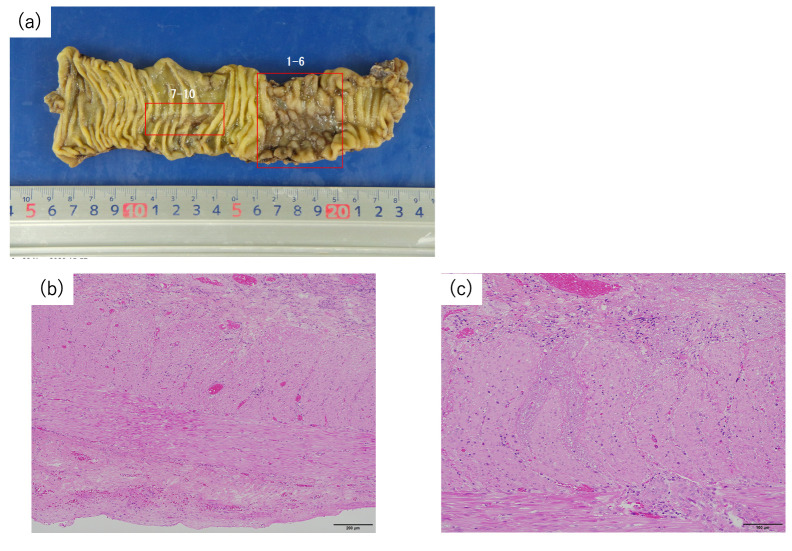
Specimen. (**a**) The irradiated intestine was ulcerated. (**b**) Fibrosis with degenerative and eosinophilic changes was observed in the muscle layer (low-power field). (**c**) In the submucosa, edema and fibrosis were revealed (high-power field).

Regarding AEs, no severe acute AEs, such as general, gastrointestinal, or skin disorders, were noted. On the other hand, three patients had sciatic neuralgia as a late AE, including two with Grade 1 and one with Grade 3. The patient with Grade 3 late AEs was prescribed nonsteroidal anti-inflammatory drugs, pregabalin, and opioids postoperatively for sciatic neuralgia. He had a limp in his left leg but could ambulate with assistance. However, no improvement in symptoms was observed during the follow-up period. The median interval between completing CIRT and surgery was four weeks (range, 3–8). The operative details and postoperative morbidities are presented in Table 3. All procedures were conducted using a laparoscopic approach. Seven patients underwent low anterior resection (LAR) with combined resection of the anastomotic site. Four patients underwent adjacent small intestine resection, and one patient underwent low anterior resection with small intestine resection. Patients who underwent LAR had diverting ileostomy. The median operating time was 313 min (range, 162–613), and the intraoperative blood loss volume was 85 mL (range, 0–1065). One patient developed Grade III pancreatitis during the mobilization of the splenic flexure, while no other patients experienced Grade III or higher postoperative complications. The duration of postoperative hospitalization was 20 days (range, 7–50). Pathology revealed severe damage to the muscle layer in the irradiated intestine, and no residual cancer cells were found (Figure 3). The median follow-up duration was 40 months (range, 20–60), and one patient was diagnosed with in-field recurrence 12 months after CIRT. The other three patients were diagnosed with out-of-field recurrence after CIRT. All patients with local re-recurrence later received reirradiation with CIRT. At the completion of the study, four patients experienced lung recurrence, and one patient died due to rectal cancer-specific causes. The 3-year overall survival (OS) rate was 90%, and the 3-year recurrence-free survival rate was 57.1%. The 3-year in-field recurrence-free survival rate was 90%, and the 3-year out-of-field recurrence-free survival rate was 72.2% (Table 4).

**Table 3:** Operative details and postoperative morbidity.

	Surgery Type	Operating Time (Minutes)	Blood Loss (mL)	Postoperative Hospitalization (Days)	Surgical Complications (C/D Grade)	Surgical Complications
1	LAR Small intestine resection	457	70	21	II	Bleeding from anastomosis
2	Small intestine resection	247	0	8	0	−
3	LAR	359	200	18	0	−
4	Small intestine resection	162	15	7	0	−
5	LAR	613	152	22	II	Urinary dysfunction
6	LAR	425	50	26	II	Ileus
7	Small intestine resection	245	1015	10	0	−
8	LAR	470	100	28	0	
9	LAR	240	0	14	0	
10	Small intestine resection	245	60	13	0	
11	LAR	267	0	30	II	Ileus
12	LAR	523	385	38	IIIa	Pancreatitis

C/D grade, Clavien–Dindo grade; LAR, low anterior resection.

**Table 4:** Oncologic outcomes of patients treated using the novel treatment strategy.

Patients	In-Field Recurrence	Time to Recurrence (Months)	Out-of-Field Recurrence	Time to Recurrence (Months)	Distant Recurrence	Time to Recurrence (Months)	Recurrence Site	Mortality	Follow-Up Duration (Months)
1	−		+	12	+	17	Lung	Yes	60
2	−		−		−		−	No	60
3	−		−		−			No	60
4	−		+	25				No	57
5	−		−		+	6	Lung	No	36
6	−		−		−		−	No	39
7	−		−		−		−	No	36
8	−		+	8	+	28	Lung	No	46
9	+	12	−		+	20	Lung	No	28
10	−		−					No	36
11	−		−		−			No	20
12	−		−		−			No	27

In-field recurrence: the enlarged tumor is inside the planned target volume. Out-of-field recurrence: the enlarged tumor is outside the planned target volume.

## 4. Discussion

In this study, we demonstrated the safety of a novel treatment strategy for patients with LRRC located less than 3 mm from the intestine, where CIRT would otherwise be contraindicated. This method involves CIRT as a curative treatment for recurrent tumors, accepting the risk of AEs affecting the adjacent intestinal tracts, followed by resection of “high-risk” irradiated intestines. Crucially, the core principle of this treatment strategy is that CIRT is not used as neoadjuvant therapy, but as the definitive curative treatment. The purpose of surgery is not tumor resection, but the removal of irradiated intestines with a high risk of severe AEs. With respect to the safety of this treatment strategy, all twelve patients did not experience severe acute AEs, but three patients experienced neuralgia as late AEs (one Grade 3 and two Grade 1), and one severe postoperative complication was deemed acceptable. With respect to the efficacy of this treatment strategy, one patient experienced in-field recurrence, and three patients experienced out-of-field recurrence. They received reirradiation with CIRT, which resulted in local control. To our knowledge, our study is the first to report the findings of this novel treatment strategy.

The advantage of our novel strategy is that full-dose CIRT provides better therapeutic effects [29]. In this study, the median tumor size was relatively small, at 24 mm. In Japan, high sacrectomy above the lower edge of the S2 vertebra is typically contraindicated, as it requires the removal of the sacroiliac joint and bilateral S1 nerves. Thus, CIRT is increasingly regarded as a promising therapeutic alternative for such patients. Prior to intestinal resection, all tumor cells had already been irradiated with curative doses, which could result in apoptosis [14]. Thus, it was hypothesized that the irradiated intestine was removed without the risk of dissemination. In a phase I/II trial, Yamada et al. revealed that irradiation at 70 Gy (RBE) or greater resulted in an overall 5-year local control rate of approximately 80% [10]. Similarly, Takiyama et al., in a cohort of 473 patients with LRRC, reported that patients without prior radiation within the pelvis (n = 390) received a dose of 73.6 Gy (RBE), whereas those with prior radiation within the pelvis (n = 83) received 70.4 Gy (RBE). The 3-year OS rates for the prior radiation-negative and prior radiation-positive groups were 73% (95% CI; 68–77%) and 76% (65–84%), respectively, with both groups achieving 3-year local control rates of 80% (75–84%) and 80% (68–88%), respectively [21]. These findings demonstrate that delivering curative doses can provide a valuable treatment option. Although CIRT achieves a high rate of local control, dose reduction is necessary when the recurrent tumor is near the intestines to minimize the risk of severe radiation-induced AEs [13]. As reported by Barcellini et al., this dose reduction resulted in approximately 80% in-field recurrence occurring at the tumor edge near the adjacent intestine. Regardless of prior RT status, a dose of at least 70.4 Gy (RBE) is essential for curative intent [24]. The results of our study suggest that our novel treatment strategy using CIRT may achieve definitive local control.

For patients with unresectable LRRC, the most important aspect is ensuring the safety of this treatment strategy. Among the AEs associated with CIRT, severe late AEs occurred in one of the twelve patients. Yamada et al. reported that acute severe AEs occurred in 10% of patients treated with CIRT, and the late severe AE rate was 21%. Therefore, these numbers of severe AEs in CIRT patients were similar to those reported in this study [22]. The patient with the severe late AE in our study developed lower limb pain, along with a left leg limp. However, this deterioration in the patient’s quality of life seemed to be within tolerance because he was able to ambulate with the assistance of a cane. Thus, we considered this case acceptable. In addition, regarding the safety of surgery for the irradiated intestine, only one patient experienced a Grade 3 complication during the postoperative course. The patient developed pancreatitis and a gastropancreatic fistula; however, endoscopic transgastric drainage was later performed successfully. Despite the low postoperative complication rates, the length of hospital stay remains long in Japan. This is because patients need to acquire stoma self-care skills before discharge due to an insufficient post-discharge support system.

The third consideration involves whether resection of the irradiated intestine is necessary. On the basis of the histopathological findings of this study, damage to muscle layers, such as fibrosis, eosinophilic changes, hyalinization, and thinning, was more severe in our patients than in those receiving conventional chemoradiotherapy (CRT). Thus, perforation of the irradiated intestines may have occurred after a certain period. Matsushita et al. reported that, compared to conventional CRT, CIRT was associated with a higher incidence of AEs occurring relatively late after irradiation (9–18 months) [23,30]. To date, the optimal period for the planned resection of the irradiated intestine is not clear. Therefore, we determined that the planned resection of the irradiated intestine within 8 weeks would provide favorable therapeutic benefits, similar to those of conventional treatment involving a long course of neoadjuvant CRT [31,32,33], thus avoiding late-onset AEs. Despite performing irradiated intestine resection within 8 weeks after the completion of CIRT, two patients exhibited severe damage to the muscle layers; therefore, early irradiated intestine resection is recommended. In addition, since we were able to transect the intestine before inflammation induced fibrosis, the removal of the irradiated intestines was not difficult. Hence, this treatment strategy is beneficial in terms of safety.

Nonetheless, our study has several limitations, mainly due to its small sample size and short observation period, and our study results are limited to the Japanese population. One patient experienced in-field recurrence, and three patients experienced out-of-field recurrence. Thus, comprehensive follow-up is needed. Moreover, further improvement of preoperative diagnostic assessments using multimodal imaging is essential to develop a better, more effective treatment strategy for LRRC patients. More data are necessary to validate the efficacy and safety of this approach.

## 5. Conclusions

In conclusion, our study revealed that a novel strategy combining CIRT followed by surgical resection of the irradiated intestine can provide favorable outcomes for patients with unresectable LRRC. This approach can represent a feasible treatment option for future patients.

## Figures and Tables

**Table 1:** Patient characteristics.

Patients	Sex	Age	RT for Primary Surgery	Primary Surgery	Histology	CRM	TNM Stage	Adjuvant CT for Primary Surgery	Recurrence Site	Reason for Nonresectability	Size of the Recurrent Tumor (mm)	Interval from Primary Surgery to Diagnosis of LRRC (Months)
1	Male	62	−	LAR	tub1	−	IIIb	CapOX	Lateral	Need to resect the S1 nerve	19	54
2	Male	68	−	APR LLND	tub1	+	IIIb	CapOX	Posterior	Need to resect the S1 nerve	16	8
3	Male	59	−	LAR	tub2	−	IIIc	CapOX	Posterior	Need to resect the S1 nerve	18	22
4	Male	38	+	LAR	tub2	+	IVc	SOX	Lateral	Need to resect the S2 nerve No consent provided for surgery	17	35
5	Male	78	−	LAR	tub2	+	IIIc	CapOX	Posterior	Need to resect the S1 nerve	29	20
6	Male	78	+	LAR	tub2	+	IIIc	Cape	Posterior	Need to resect the S1 nerve	26	16
7	Male	66	+	APR LLND	tub1	−	I	−	Lateral	Difficult to secure the lateral margin	30	9
8	Female	51	−	LAR	tub2	−	IV	CapOX	Posterior	Need to resect the S1 nerve	37	34
9	Male	69	−	LAR	tub2	−	IIIb	Cape	Lateral	Need to resect the S1 nerve	22	36
10	Male	44	+	LAR	tub2	−	I	−	Lateral	Need to resect the S1 nerve	22	12
11	Female	67	−	LAR	tub2	−	IIIc	CapOX	Posterior	Need to resect the S1 nerve	29	93
12	Male	50	−	LAR	por2	+	IIIc	CapOX	Posterior	Need to resect the S1 nerve	25	23

RT, radiation therapy; CT, chemotherapy; LRRC: locally recurrent rectal cancer; LAR, low anterior resection; APR, abdominoperineal resection; LLND, lateral lymph node dissection; CapeOX, oxaliplatin and capecitabine; SOX, oxaliplatin and S-1.

**Table 2:** Details of patients who received CIRT.

Patients	Dose (Gy)	Waiting Time Before Surgery (Weeks)	Acute AEs	Late AEs	Time to Onset (Months)	Histological Findings
1	73.6	8	None	Sciatic nerve pain Grade 3	9	No presence of cancer cells
2	73.6	5	None	None		No presence of cancer cells
3	73.6	8	None	None		No presence of cancer cells
4	70.4	4	None	Sciatic nerve pain Grade 1	6	No presence of cancer cells
5	73.6	6	None	Sciatic nerve pain Grade 1	15	No presence of cancer cells
6	73.6	4	None	None		No presence of cancer cells
7	70.4	2	None	None		No presence of cancer cells
8	73.6	3	None	None		No presence of cancer cells
9	73.6	3	None	None		No presence of cancer cells
10	70.4	3	None	None		No presence of cancer cells
11	73.6	8	None	None		No presence of cancer cells
12	73.6	5	None	None		No presence of cancer cells

CIRT, carbon ion radiotherapy; AEs, adverse events.

## Data Availability

The data used in this study are available upon request from the corresponding authors on the ethically approved research protocol.

## Abbreviations

The following abbreviations are used in this manuscript:

CIRT	Carbon-ion radiotherapy
LRRC	Locally recurrent rectal cancer
RBE	Relative biologic effectiveness
AEs	Adverse events
QST	Quantum Science and Technology
CEA	Carcinoembryonic antigen
CA19-9	Carbohydrate antigen
CT	Computed tomography
MRI	Magnetic resonance imaging
LAR	Low anterior resection
